# Outcomes of GLP-1 receptor agonist therapy in adults with sickle cell disease and type 2 diabetes: a real-world cohort analysis

**DOI:** 10.1186/s13023-026-04322-5

**Published:** 2026-03-28

**Authors:** Guy Loic Nguefang, Sarpong Boateng, Solomon Gyabaah, Yussif Issaka, Joel Gabin Konlack, Edgar Luna Landa, Elvis Eze, Basile Njei

**Affiliations:** 1 Texas Tech University Health Sciences Center, Odessa, TX USA; 2 Yale New Haven Health, Bridgeport Hospital, New Haven, Connecticut, USA; 3 Komfo Anokye Teaching Hospital, Kumasi, Ghana; 4 New York City Health and Hospitals South Brooklyn Health, Brooklyn, NY USA; 5 Yale University, New Haven, Connecticut, USA; 6 Engelhardt School of Global Health and Bioethics, Euclid University, Bangui, Central African Republic; 7 Artificial Intelligence Program, University of Cumbria, Carlisle, UK; 8 Ohio University Heritage College of Osteopathic Medicine, Athens, OH USA; 9 Yale Liver Center, Yale New Haven Health, New Haven, Connecticut, USA; 10 Yale International Medicine Program, Yale University, New Haven, Connecticut, USA

**Keywords:** Sickle cell disease, Type 2 diabetes mellitus, Glucagon-like peptide-1 receptor agonist (GLP-1), Major adverse cardiovascular events (MACE), Vaso-occlusive crises

## Abstract

**Purpose:**

Outcomes of patients with sickle cell disease (SCD) and type 2 diabetes mellitus (T2DM) treated with glucagon-like peptide-1 (GLP1-RA) remain poorly understood. This study evaluated the impact of GLP-1 on adults with SCD and T2DM beyond glycemic control.

**Methods:**

We conducted a retrospective study within the TriNetX Network, identifying adults with SCD and T2DM. GLP-1 users and non–GLP-1 users were matched using Propensity score matching. The index date was defined as the first GLP-1 prescription, and outcomes were assessed over 12 months. Outcomes were major adverse cardiovascular events (MACE), chronic kidney disease (CKD), vaso-occlusive crises (VOC), and hypoglycemia. Hazard ratios (HRs) with 95% confidence intervals (CIs) were estimated using Cox proportional hazards models.

**Results:**

We included 1,391 patients in each cohort, with similar age (48.5 vs. 48.8), and most participants were Black (84.5% vs. 83.8%). GLP-1 therapy was associated with lower risk of MACE (HR 0.61, 95% CI 0.49–0.76; *P* < 0.00001) and a reduced risk of stroke (HR 0.47, 95% CI 0.23–0.97; *P* = 0.036). Additionally, GLP-1 use was linked to a 36% lower risk of VOC (HR 0.64, 95% CI 0.45–0.91; *P* = 0.013). There was no difference in risk of hypoglycemia (HR 1.10, 95% CI 0.60–1.76; *P* = 0.688) or progression to CKD between the two groups (HR 1.26, 95% CI 0.82–1.95; *P* = 0.294).

**Conclusions:**

GLP-1 therapies demonstrate favorable cardioprotective, vaso stabilizing, and safety benefits in adults with SCD and T2DM, supporting their use in this high-risk population.

**Clinical trial number:**

Not applicable.

**Supplementary Information:**

The online version contains supplementary material available at 10.1186/s13023-026-04322-5.

## Introduction

Sickle cell disease (SCD) is an inherited hemoglobinopathy characterized by chronic intravascular hemolysis, systemic inflammation, endothelial dysfunction, and recurrent vaso-occlusive events, all of which contribute to substantial morbidity and mortality [1, 2]. Globally, SCD affects an estimated 7.7 million individuals, representing a major public health challenge. In the United States alone, approximately 100,000 people live with SCD, and the disease remains associated with a reduction in life expectancy of nearly 30 years [3]. Advances in newborn screening, supportive care, and emerging disease-modifying therapies have significantly improved survival, leading to a growing adult SCD population [1, 4]. As patients live longer, the clinical burden has shifted from predominantly acute complications toward chronic cardiovascular, renal, and metabolic comorbidities now acting as drivers of long-term morbidity and premature mortality [4, 5].

The coexistence of type 2 diabetes mellitus (T2DM) with SCD represents a particularly high-risk clinical phenotype. Diabetes further exacerbates the pre-existing cardiovascular, renal, and neurovascular vulnerabilities inherent to SCD, compounding the overall disease burden [6, 7]. Poorly controlled diabetes can markedly worsen clinical outcomes in adults with SCD, underscoring the need for antidiabetic therapies that not only improve glycemic control but also target the synergistic cardiometabolic risks present in this population.

Glucagon-like peptide-1 receptor agonists (GLP-1 RAs) are established therapies for T2DM with benefits that extend beyond glycemia. Large cardiovascular outcome trials have demonstrated that GLP-1 RAs significantly reduce cardiovascular and all-cause mortality, while also improving body weight, blood pressure, systemic inflammation, and endothelial function [8–10]. Despite these broad metabolic and protective effects in diverse high-risk populations, GLP-1 RAs have not been systematically evaluated in individuals with SCD, a population in whom optimizing vascular and metabolic health may have substantial clinical impact.

To address this critical evidence gap, we conducted a retrospective cohort study using the TriNetX Research Network. Our objective was to assess cardiovascular, renal, and SCD-related outcomes among adults living with both SCD and T2DM.

## Methodology

### Study design and data source

We conducted a retrospective cohort study using data from the TriNetX Research Network, a federated platform that aggregates de-identified electronic health records (EHRs) from approximately 70 U.S. healthcare organizations. TriNetX provides standardized information on demographics, diagnoses, procedures, medications, and laboratory values, enabling real-time queries and analytics while ensuring compliance with HIPAA and institutional privacy regulations. Cohort construction and analyses were performed within the TriNetX analytics platform, and the study adhered to the Strengthening the Reporting of Observational Studies in Epidemiology (STROBE) guidelines.

### Study population

We included adults aged 18 years or older with a documented history of SCD and T2DM. SCD patients were identified using ICD-10 diagnostic (Table S1). Patients were excluded if they had type 1 diabetes mellitus; other specified, drug-induced, or secondary forms of diabetes; end-stage renal disease; prior solid organ transplantation; malignancy; pregnancy; gastrointestinal motility disorders; or a history of bariatric surgery.

Two mutually exclusive cohorts were defined based on exposure to GLP-1 RAs (semaglutide, liraglutide, dulaglutide, exenatide, lixisenatide, and tirzepatide). The GLP-1 RA cohort included SCD patients with T2DM who were initiated on GLP-1 RA therapy after their initial diagnosis and had at least two documented prescriptions. The comparison cohort comprised SCD patients with T2DM who received conventional diabetes treatment (insulin and/or oral hypoglycemic agents) without any documented exposure to GLP-1RAs.

### Index event and follow-up window

The index date was defined separately for each cohort. For GLP-1 RA users, the index date corresponded to the earliest encounter at which both SCD diagnosis and GLP-1 RA prescription were documented. For non-users, the index date was the earliest SCD diagnosis in the absence of prior or subsequent GLP-1 RA exposure. Outcomes were evaluated, beginning one day after the index date and continuing through 12 months.

### Outcomes

The primary outcome was the occurrence of major adverse cardiovascular events (MACE) which included heart failure, cardiac arrest, myocardial infarction, atrial fibrillation, atrial flutter, and stroke. Secondary outcomes included progression to CKD, Vaso-occlusive crises (VOC), hypoglycemia, and health care system utilization (hospital visits or admissions). Outcomes were identified using ICD-10 diagnostic codes and standardized TriNetX definitions. To ensure accurate risk attribution, patients with a documented history of any outcome prior to the index date were excluded from the corresponding analysis.

We acknowledge that this expanded MACE definition differs from the conventional 3-point or 4-point MACE composites used in cardiovascular outcome trials. This broader definition was adopted to capture the full spectrum of cardiovascular events pertinent to SCD and because cardiovascular death, a component of standard MACE definitions, could not be reliably ascertained in the TriNetX database.

### Propensity score matching

To minimize confounding and achieve balanced baseline characteristics between cohorts, we performed 1:1 propensity score matching (PSM) using the TriNetX matching algorithm. Matching variables included demographic factors (age, sex, race, ethnicity), clinical comorbidities (obesity, hypertension, hyperlipidemia, CKD, cerebral infaction, and SCD genotype), and medications (hydroxyurea, antilipemic agents). Clinically important covariates including SCD genotype, prior VOC frequency, could not be reliably captured through the TriNetX platform and were therefore not included in the propensity score model. Covariate balance was evaluated using standardized mean differences, with values less than 0.1 considered indicative of adequate balance.

### Statistical analysis

All statistical analyses were conducted within the TriNetX analytics platform. Categorical outcomes were evaluated using measures of association, including risk ratios. Time-to-event analyses were performed using Kaplan–Meier survival curves and hazard ratio estimation with corresponding 95% confidence intervals. Statistical significance was defined as a two-sided p-value < 0.05.

### Ethical considerations

This study utilized fully de-identified, aggregated patient-level data from the TriNetX Research Network. Because no identifiable private information was accessed and all analyses were conducted within a secure, privacy-preserving environment, the study qualified for exemption from institutional review board oversight.

## Results

### Baseline characteristics of study participants

After propensity score matching, 1,391 patients were included in each cohort, with well-balanced baseline characteristics. The mean age was similar between the GLP-1 RA cohort and the non–GLP-1 RA cohort (48.5 ± 13.7 vs. 48.8 ± 15.8 years, standardized difference [Std Diff] = 0.018; *P* = 0.627). Sex distribution was comparable between groups, with females comprising 72.4% of the GLP-1 RA cohort and 74.0% of the non–GLP-1 RA cohort. Most participants were Black or African American, with similar proportions in both cohorts (84.5% vs. 83.8%, Std Diff = 0.020; *P* = 0.603). Other racial and ethnic groups were evenly distributed, including White (6.0% vs. 6.6%), Asian (0.9% vs. 0.8%), American Indian or Alaska Native (0.7% vs. 0.7%), Native Hawaiian or Pacific Islander (0.7% vs. 0.7%), and other or unknown race (5.8% vs. 5.2%).

Baseline comorbidities were comparable between the two cohorts. Rates of hyperlipidemia were similar between GLP-1 RA users and non-users (37.7% vs. 37.6%), as were obesity (44.4% vs. 44.5%), hypertensive diseases (52.9% vs. 51.5%), CKD (8.9% vs. 9.4%), and prior cerebral infarction (3.7% vs. 3.6%). Medication use was also well balanced, with comparable proportions receiving antilipemic agents (31.9% vs. 31.8%) and hydroxyurea (1.9% vs. 2.0%). Laboratory and anthropometric measures were similar between groups. Mean body mass index was comparable (38.1 ± 9.5 vs. 37.6 ± 8.9 kg/m², Std Diff = 0.055; *P* = 0.259), with nearly identical distributions across BMI categories. Mean HbA1c was higher in the GLP-1 RA cohort (7.7 ± 2.3% vs. 7.0 ± 1.8%, Std Diff = 0.368; *P* < 0.001). All data on population characteristics are presented in Table 1 and Table S2; Propensity score density distribution in Figure S1.

### Primary outcome

#### MACE

After propensity score matching, MACE occurred in 10.4% of patients in the GLP-1 RA cohort compared with 16.0% in the non GLP-1 RA cohort. GLP-1 RA use was associated with a 39% lower risk of MACE (HR 0.61, 95% CI 0.49–0.76; *P* < 0.00001) (Table 2).

### Cardiovascular subgroup outcomes

#### Stroke

Stroke occurred in 0.8% of GLP-1 RA users compared with 1.8% of non-users. GLP-1 RA therapy was associated with a 53% reduction in stroke risk (HR 0.47, 95% CI 0.23–0.97; *P* = 0.036) (Table 2).

#### Heart failure

Heart failure occurred in 1.7% of GLP-1 RA users versus 2.6% of non-users. Although the point estimate favored GLP-1 RA use, this difference did not reach statistical significance (HR 0.63, 95% CI 0.36–1.10; *P* = 0.105) (Table 2).

### Secondary outcomes

#### VOC

VOC occurred in 3.6% of patients treated with GLP-1 RAs compared with 5.5% in the non-GLP-1 RA cohort. GLP-1 RA use was associated with a 36% lower risk of VOC (HR 0.64, 95% CI 0.45–0.91; *P* = 0.013) (Table 2).

#### CKD

Incident CKD occurred in 3.7% of GLP-1 RA users and 2.9% of non-users. There was no statistically significant difference in CKD risk between groups (HR 1.26, 95% CI 0.82–1.95; *P* = 0.294) (Table 2).

#### Hypoglycemia

Hypoglycemia occurred in 2.7% of GLP-1 RA users compared with 2.4% of non-users, with no significant difference between cohorts (HR 1.10, 95% CI 0.60–1.76; *P* = 0.688) (Table 2).

#### Hospital visits or admissions

All-cause hospital visits or admission occurred in 19% of GLP-1 RA users versus 40% of non-users. GLP-1 RA therapy was associated with a reduced risk of hospital visits/admissions (HR 0.39, 95% CI 0.33–0.47; *p* < 0.0001) (Table 2).

**Table 1:** Baseline characteristics of adults with sickle cell disease and type 2 diabetes mellitus after propensity score matching

Characteristic	GLP-1 RA Cohort (*N* = 1,391) [n (%)]	Non-GLP-1 RA Cohort (*N* = 1,391) [n (%)]	Std Diff	*P*-value
Age, years (mean ± SD)	48.5 ± 13.7	48.8 ± 15.8	0.02	0.627
Female	1,007 (72.4)	1,029 (74.0)	0.04	0.346
Male	382 (27.5)	361 (26.0)	0.03	0.368
**Race / Ethnicity**				
White	84 (6.0)	92 (6.6)	0.02	0.533
Black / African American	1,176 (84.5)	1,166 (83.8)	0.02	0.603
Asian	12 (0.9)	11 (0.8)	0.008	0.834
American Indian / Alaska Native	10 (0.7)	10 (0.7)	< 0.001	1.000
Native Hawaiian / Pacific Islander	10 (0.7)	10 (0.7)	< 0.001	1.000
Other / Unknown race	80 (5.8)	73 (5.2)	0.02	0.560
**Comorbidities**				
Hyperlipidemia	525 (37.7)	523 (37.6)	0.003	0.938
Obesity	618 (44.4)	619 (44.5)	0.001	0.970
Hypertensive diseases	736 (52.9)	717 (51.5)	0.027	0.471
Chronic kidney disease	124 (8.9)	131 (9.4)	0.017	0.646
Cerebral infarction	51 (3.7)	50 (3.6)	0.004	0.919
**Medications**				
Antilipemic agents	444 (31.9)	443 (31.8)	0.002	0.968
Hydroxyurea	26 (1.9)	28 (2.0)	0.01	0.783
**Laboratory Values**				
HbA1c, % (mean ± SD)	7.7 ± 2.3	7.0 ± 1.8	0.37	< 0.001
HbA1c ≤ 5.7%	163 (11.7)	157 (11.3)	0.01	0.721
HbA1c 5.8–6.4%	340 (24.4)	341 (24.5)	0.002	0.965
HbA1c ≥ 6.5%	591 (42.5)	584 (42.0)	0.01	0.788
**Body Mass Index (BMI)**	38.1 ± 9.5	37.6 ± 8.9	0.05	0.259
0–18.50 kg/m²	17 (1.2)	18 (1.3)	0.006	0.865
18.50–24.90 kg/m²	69 (5.0)	56 (4.0)	0.04	0.234
25–29.90 kg/m²	207 (14.9)	206 (14.8)	0.002	0.957
30–34.90 kg/m²	296 (21.3)	305 (21.9)	0.02	0.678
35–39.90 kg/m²	289 (20.8)	295 (21.2)	0.01	0.780
≥ 40 kg/m²	362 (26.0)	362 (26.0)	< 0.001	1.000

Baseline demographic, clinical, medication, and laboratory characteristics of adults with sickle cell disease and type 2 diabetes mellitus treated with glucagon-like peptide-1 receptor agonists (GLP-1 RA cohort) and those not receiving GLP-1 RA therapy (non-GLP-1 RA cohort) after 1:1 propensity score matching. Values are presented as mean ± standard deviation or number (percentage). Standardized differences (Std Diff) are reported to assess covariate balance between cohorts, with values < 0.10 indicating adequate balance

**Table 2:** Clinical outcomes in adults with sickle cell disease and type 2 diabetes mellitus treated with and without GLP-1 RA

Outcomes	GLP-1 RA Cohort (*N* = 1,391) (%)	Non-GLP-1 RA Cohort (*N* = 1,391) (%)	HR (95% CI)	*P* value
MACE	10.4	16	0.61 (0.49–0.76)	< 0.00001 *
Stroke	0.8	1.8	0.47 (0.23–0.97)	0.036 *
Heart Failure	1.7	2.6	0.63 (0.36–1.10)	0.105
CKD	3.7	2.9	1.26 (0.82–1.95)	0.294
Vaso Occlusion Crisis	3.6	5.5	0.64 (0.45–0.91)	0.013 *
Hypoglycemia	2.7	2.4	1.10 (0.60–1.76)	0.688
Hospital visits or admissions	19	40	0.39 (0.33–0.47)	0.0001*

Comparison of cardiovascular, renal (chronic kidney disease), sickle cell disease–related, and glycemic outcomes between adults with sickle cell disease and type 2 diabetes mellitus receiving glucagon-like peptide-1 receptor agonist (GLP-1 RA cohort) therapy and matched non–GLP-1 RA users over one year of follow-up. Hazard ratios (HRs) with 95% confidence intervals (CIs) were estimated using Cox proportional hazards models after propensity score matching. Asterisks (*) denote statistically significant associations (*P* < 0.05)

## Discussion

In this cohort study of adults with SCD and T2DM, GLP-1 RA use was associated with a significant reduction in MACE over one year of follow-up. Beyond this cardiovascular benefit, GLP-1 RA therapy was also linked to a significantly lower risk of stroke and a reduced incidence of VOC. Notably, these advantages occurred without an increased risk of hypoglycemia. In contrast, no significant differences were observed between cohorts in rates of heart failure or progression to CKD.

Cardiovascular disease is a major contributor to morbidity and mortality in adults with SCD. In our cohort, GLP-1 RA therapy was associated with a 39% reduction in MACE and a 53% reduction in stroke, findings that exceed the reductions of 13% typically reported in the general T2DM population [8, 11]. These benefits occurred despite higher baseline hemoglobin A1c levels in GLP-1 RA users, supporting the notion that GLP-1 RA –mediated cardiovascular protection extends beyond glycemic control [8]. The observed effects likely reflect GLP-1 RA ’s anti-inflammatory, endothelial-protective, and antithrombotic actions. Taken together, the reductions in MACE and stroke underscore the potential value of integrating GLP-1 RA therapy into the management of adults with SCD and T2DM to improve cardiovascular outcomes.

The 36% reduction in VOC associated with GLP-1 RA therapy represents a novel and potentially practice-changing finding. This association suggests that GLP-1 RA may interrupt the vascular and inflammatory cascade underlying VOC through mechanisms distinct from current sickle cell disease–specific therapies [5, 12]. GLP-1 RA signaling modulates several pathways central to VOC pathophysiology, including attenuation of endothelial dysfunction, inhibition of oxidative stress, suppression of pro-inflammatory cytokine signaling, and enhancement of nitric oxide bioavailability and microvascular perfusion [13–15]. Moreover, the metabolic and vascular stabilization conferred by GLP-1 RA therapy may reduce VOC frequency [10, 16]. Although the reduction in VOC is biologically plausible, alternative explanations must also be considered. Patients prescribed GLP 1 therapy may have more consistent access to care, closer clinical monitoring, and greater adherence to supportive treatments such as hydration and hydroxyurea. Differences in health-related behaviors including higher health literacy, greater engagement with the healthcare system, and more effective self-management of SCD triggers may likewise contribute to the observed benefit. While PSM helped balance measurable confounders, unmeasured differences in health seeking behavior and care intensity between cohorts cannot be excluded.

The difference in progression to CKD between the two cohorts was not statistically significant. This finding is consistent with the distinct renal pathophysiology of SCD, in which sickle nephropathy begins early in life with medullary ischemia and glomerular hyperfiltration, leading to cumulative microvascular injury and progressive, irreversible renal damage—limiting the capacity of newer interventions to alter long-term trajectories [17]. Although GLP-1 RA have demonstrated renal benefits in non-SCD populations, these effects are largely driven by reductions in albuminuria rather than prevention of advanced kidney failure. Taken together, the absence of a significant CKD effect in this SCD cohort likely reflects the unique, multifactorial progression of sickle nephropathy rather than a lack of therapeutic potential of GLP-1 RA agents [18, 19].

The finding that hypoglycemia occurred at similar rates among GLP-1 RA users and non-users provides reassuring evidence regarding the safety of GLP-1 RA therapy in SCD. This observation is particularly relevant because HbA1c levels remained modestly imbalanced between our groups. As glycemic control may influence VOC and other complications, residual confounding related to baseline HbA1c cannot be excluded. However, interpretation of HbA1c in SCD is inherently challenging, as altered red blood cell turnover can render HbA1c values unreliable or difficult to interpret [20, 21]. In this context of imprecise glycemic monitoring, the demonstration of cardiovascular and vaso-occlusive benefits without a difference in hypoglycemia supports GLP-1 RA as a safe therapeutic option than traditional glucose-lowering agents for patients with SCD and T2DM, in whom avoiding hypoglycemia is especially critical.

### Clinical implications

Taken together, these findings provide supportive real-world evidence that GLP-1 RA may offer cardiovascular, vascular, and safety benefits in adults with SCD and T2DM. While these data do not establish causality, they suggest that GLP-1 RA therapy warrants further evaluation as an effective therapeutic option in this high-risk population.GLP-1 RA. Notably, the cardiovascular benefits including reductions in MACE and stroke appear early after initiation and extend beyond weight loss or glycemic control.

### Strengths

This study has several strengths. First, it leverages a large, national, real-world cohort derived from a federated electronic health record network, enhancing generalizability across diverse healthcare settings in the United States. Second, the use of propensity score matching allowed for robust balancing of demographic characteristics, comorbidities, and concomitant medications, thereby mitigating confounding and strengthening the validity of observed associations. Third, this study addresses a critical knowledge gap by evaluating GLP-1 RA therapy in adults with SCD, who present unique cardiometabolic and vascular vulnerability. Finally, the use of standardized, longitudinal electronic health record data enabled consistent outcome ascertainment and real-world assessment of treatment effectiveness beyond the constraints of randomized trials.

### Limitations

Several limitations should be considered. The retrospective observational design precludes causal inference, and residual confounding may persist despite rigorous propensity score matching. Additionally, outcomes were identified using ICD-10 codes within electronic health records, which may introduce misclassification bias.

Another important limitation is the differential definition of the index date between cohorts. GLP-1 RA users entered follow-up at the time of treatment initiation, whereas non-users were indexed at the earliest SCD diagnosis, potentially introducing non-comparable baseline risk periods and immortal time bias as GLP-1 RA users may have had longstanding SCD prior to therapy initiation. To partially mitigate this concern, patients with a documented history of any studied outcome prior to the index date were excluded from the corresponding outcome analysis, thereby restricting events to incident occurrences during the follow-up window. However, this approach does not fully eliminate the potential for temporal misalignment between cohorts, and this limitation should be considered when interpreting the observed associations.

Additionally, GLP-1 RA use was treated as a fixed baseline exposure, which does not capture the inherently time-dependent nature of medication use including delayed initiation, dose changes, or discontinuation during follow-up. The TriNetX platform lacks granular longitudinal prescription data necessary to implement time-varying exposure models. Future studies with pharmacy claims or electronic prescribing data capable of capturing treatment dynamics would more accurately characterize GLP-1 RA exposure over time.

GLP-1 RA prescriptions are influenced by socioeconomic factors, insurance coverage, formulary status, and access to specialty care factors that are not captured in the TriNetX database. Patient prescribed GLP-1 RA therapy may therefore differ systematically from non-users in ways that are not fully addressed by PSM, including greater engagement with the healthcare system, higher health literacy, or insurance coverage. This potential confounding by access and health-seeking behavior should be considered when interpreting the observed associations.

The 12-month follow-up period was selected to maximize cohort retention and outcome ascertainment within the constraints of a real-world EHR database. However, this duration is likely insufficient to detect differences in slowly progressive outcomes such as CKD, which in SCD evolves through cumulative medullary ischemia and glomerular hyperfiltration over years to decades. The absence of a statistically significant CKD effect should therefore be interpreted with this temporal constraint in mind, and longer-term studies are needed to evaluate the renal effects of GLP-1 RA therapy in SCD.

VOC were identified using ICD-10 codes. This claims-based approach captures VOC episodes that result in healthcare encounters (emergency department visits or hospitalizations) but may underestimate true VOC burden, as crises managed at home or treated in outpatient settings without specific diagnostic coding would not be captured. This ascertainment bias likely applies equally to both cohorts and would, if anything, attenuate the observed treatment effect. Despite these limitations, the consistency of findings across multiple clinically meaningful outcomes supports the robustness of the observed associations.

### Future directions

These findings underscore the need for prospective studies to validate the observed associations in adults with SCD and T2DM. Future investigations should include longer follow-up periods to assess the durability of benefit particularly for renal outcomes and prioritize mechanistic studies to clarify how GLP-1 RA influence VOC. Randomized trials comparing GLP-1 RA therapy with insulin-based regimens in SCD populations are also necessary to define comparative effectiveness and safety in real-world clinical practice. We acknowledge that other adverse effects of GLP 1 therapy may be of greater relevance in this population. Gastrointestinal side effects (such as nausea and vomiting), sarcopenia associated with rapid GLP 1–mediated weight loss, and the known risks of pancreatitis and cholecystitis warrant dedicated evaluation in future studies of GLP 1 therapy in SCD populations. Given the heightened intrinsic risk of venous thromboembolism (VTE) in individuals with SCD, future studies should also specifically assess VTE as a safety endpoint in this population. Such work will be essential to inform evidence-based guidelines and optimize integrated cardiometabolic and hematologic care for adults living with SCD.

## Conclusion

In this cohort of adults with SCD and T2DM, GLP-1 RA therapy was associated with significant reductions in MACE, stroke, and VOC without an increased risk of hypoglycemia These findings contribute supportive real-world evidence regarding the potential benefit and safety profile of GLP-1 RA in this population. While the observational nature of this study precludes causal inference, the consistency of favorable associations across cardiovascular, hematologic, and safety outcomes supports the need for prospective studies and randomized trials.

## Supplementary Information

Below is the link to the electronic supplementary material.

Supplementary Material 1

## Data Availability

The data underlying this study are derived from a licensed national healthcare database. Access to the dataset requires a data-use agreement with the data provider. Aggregate data and analytic code used to generate the findings are available in the supplementary file.

## Declarations

### Assistance with the study

No external assistance was sought or received for the design, execution, analysis, or reporting of this study.

### Presentation

This work has not been previously presented elsewhere.
