# Supplementary material for: Outcomes of GLP-1 receptor agonist therapy in adults with sickle cell disease and type 2 diabetes: a real-world cohort analysis
Source: Orphanet J Rare Dis. 2026 Mar 28;21:126. doi: 10.1186/s13023-026-04322-5 (PMC13047803; doi:10.1186/s13023-026-04322-5)
Supplement: Supplementary file 1 — Supplementary Material 1 [file 13023_2026_4322_MOESM1_ESM.docx]

**Supplementary file**

**Table S1**. Diagnostic, Medication, and Procedure Codes Used for Cohort Definition

| **Domain** | **Code Type** | **Code** | **Description** |
| --- | --- | --- | --- |
| Inclusion criteria | | | |
|  | ICD-10 | E11 | Type 2 diabetes mellitus |
|  | ICD-10 | D57 | Sickle cell disorders |
| Medication | RxNorm | 1991302 | Semaglutide |
|  | RxNorm | 475968 | Liraglutide |
|  | RxNorm | 60548 | Exenatide |
|  | RxNorm | 1534763 | Albiglutide |
|  | RxNorm | 1551291 | Dulaglutide |
|  | RxNorm | 1440051 | Lixisenatide |
|  | VA Drug Class | HS502 | Oral hypoglycemic agents |
|  | VA Drug Class | HS501 | Insulin |
| Outcomes | Vaso-occlusive crisis (VOC) | D57.0 | Hb-SS disease with crisis |
|  |  | D57.2 | Double heterozygous sickling disorders with crisis |
|  | Chronic Kidney Disease | N18 | Chronic kidney disease |
|  | Hypoglycemia | E16.0 | Drug-induced hypoglycemia |
|  |  | E16.1 | Other hypoglycemia |
|  |  | E16.2 | Hypoglycemia, unspecified |
| Healthcare Utilization Outcomes | Emergency department visit | — | Encounter type classification in TriNetX |
|  | Hospitalization | — | Inpatient encounter classification |
|  | ICU admission | — | ICU-level encounter classification |
| Propensity Score Matching Covariates | Hypertension | I10 |  |
|  | Ischemic heart disease | I20–I25 |  |
|  | Heart failure | I50 |  |
|  | Dyslipidemia | E78 |  |
|  | Obesity | E66 |  |
|  | Chronic kidney disease | N18 |  |
|  | MACE | I63, I50, I21, I22, I63, I61, I60, I46, I50, I48 |  |
| Exclusion criteria | | | |
|  | ICD-10 | E08* | Diabetes mellitus due to underlying condition (all variants) |
|  | ICD-10 | E09* | Drug- or chemical-induced diabetes mellitus (all variants) |
|  | ICD-10 | E10* | Type 1 diabetes mellitus (all variants) |
|  | ICD-10 | E13* | Other specified diabetes mellitus (all variants) |
|  | ICD-10 | N18.6 | End-stage renal disease |
|  | ICD-10 | C00–D49 | Neoplasms |
|  | ICD-10 | Z94.0 | Kidney transplant status |
|  | ICD-10 | Z94.1 | Heart transplant status |
|  | ICD-10 | Z94.2 | Lung transplant status |
|  | ICD-10 | Z94.4 | Liver transplant status |
|  | ICD-10 | Z33.1 | Pregnant state, incidental |
|  | ICD-10 | K56* | Paralytic ileus and intestinal obstruction (all variants) |
|  | ICD-10 | K31.84 | Gastroparesis |
|  | HCPCS | E0860 | Traction equipment, overdoor, cervical |
|  | CPT | 43644 | Laparoscopic gastric bypass with Roux-en-Y |
|  | CPT | 43775 | Laparoscopic sleeve gastrectomy |

*Abbreviations:* ICD-10, International Classification of Diseases, Tenth Revision; RxNorm, normalized drug nomenclature; CPT, Current Procedural Terminology; HCPCS, Healthcare Common Procedure Coding System.
*Asterisk (*) indicates inclusion of all subcodes. *

**Table S2.** Baseline Characteristics of Adults with Sickle Cell Disease and Type 2 Diabetes Mellitus Before Propensity Score Matching

| **Characteristic** | **GLP-1 Cohort (N = 1,729)** | **Non-GLP-1 Cohort (N = 5,244)** | **Std Diff** | **P-value** |
| --- | --- | --- | --- | --- |
| Age, years (mean ± SD) | 48.8 ± 13.6 | 50.1 ± 16.1 | 0.090 | 0.002 |
| Female | 1,284 (74.3%) | 3,347 (64.4%) | 0.214 | <0.001 |
| Male | 443 (25.6%) | 1,843 (35.5%) | 0.215 | <0.001 |
| **Race / Ethnicity** | | | | |
| White | 100 (5.8%) | 402 (7.7%) | 0.078 | 0.007 |
| Black / African American | 1,470 (85.0%) | 4,317 (83.1%) | 0.052 | 0.064 |
| Asian | 18 (1.0%) | 36 (0.7%) | 0.038 | 0.154 |
| American Indian / Alaska Native | 10 (0.6%) | 13 (0.3%) | 0.051 | 0.040 |
| Native Hawaiian / Pacific Islander | 10 (0.6%) | 10 (0.2%) | 0.062 | 0.010 |
| Other / Unknown race | 94 (5.4%) | 275 (5.3%) | 0.006 | 0.820 |
| **Comorbidities** | | | | |
| Hyperlipidemia | 775 (44.8%) | 1,135 (21.9%) | 0.502 | <0.001 |
| Obesity | 889 (51.4%) | 1,114 (21.4%) | 0.655 | <0.001 |
| Hypertensive diseases | 989 (57.2%) | 2,009 (38.7%) | 0.377 | <0.001 |
| Chronic kidney disease | 169 (9.8%) | 386 (7.4%) | 0.084 | 0.002 |
| Cerebral infarction | 61 (3.5%) | 223 (4.3%) | 0.039 | 0.165 |
| **Medications** |  |  |  |  |
| Antilipemic agents | 718 (41.5%) | 734 (14.1%) | 0.642 | <0.001 |
| Hydroxyurea | 29 (1.7%) | 165 (3.2%) | 0.098 | 0.001 |
| **Laboratory Values** | | | | |
| HbA1c, % (mean ± SD) | 7.9 ± 2.3 | 6.7 ± 1.7 | 0.559 | <0.001 |
| HbA1c ≤ 5.7% | 202 (11.7%) | 377 (7.3%) | 0.152 | <0.001 |
| HbA1c 5.8–6.4% | 469 (27.1%) | 730 (14.1%) | 0.328 | <0.001 |
| HbA1c ≥ 6.5% | 907 (52.5%) | 891 (17.2%) | 0.798 | <0.001 |
| **Body Mass Index (BMI)** | 38.6 ± 9.3 | 34.3 ± 8.9 | 0.477 | <0.001 |
| 0 - 18.50 kg/m² | 19 (1.1%) | 83 (1.6%) | 0.043 | 0.136 |
| 18.50 - 24.90 kg/m² | 71 (4.1%) | 409 (7.9%) | 0.159 | <0.001 |
| 25 - 29.90 kg/m² | 250 (14.5%) | 749 (14.4%) | 0.001 | 0.968 |
| 30 - 34.90 kg/m² | 391 (22.6%) | 856 (16.5%) | 0.155 | <0.001 |
| 35 - 39.90 kg/m² | 415 (24.0%) | 629 (12.1%) | 0.313 | <0.001 |
| ≥ 40 kg/m² | 509 (29.4%) | 674 (13.0%) | 0.411 | <0.001 |

Baseline demographic, clinical, medication, and laboratory characteristics of adults with sickle cell disease and type 2 diabetes mellitus treated with glucagon-like peptide-1 receptor agonists (GLP-1 cohort) and those not receiving GLP-1 therapy (non-GLP-1 cohort) prior to propensity score matching. Values are presented as mean ± standard deviation or number (percentage). Standardized differences (Std Diff) and *P* values are shown to illustrate baseline imbalance between cohorts, with larger standardized differences indicating substantial differences in covariate distribution before matching.

**Figure S1.** Propensity Score Density Distributions Before and After Matching

| **Propensity score density function - Before and after matching (SCD with GLP1 - purple, SCD without GLP1 - green)** | | | |
| --- | --- | --- | --- |
|  |  | 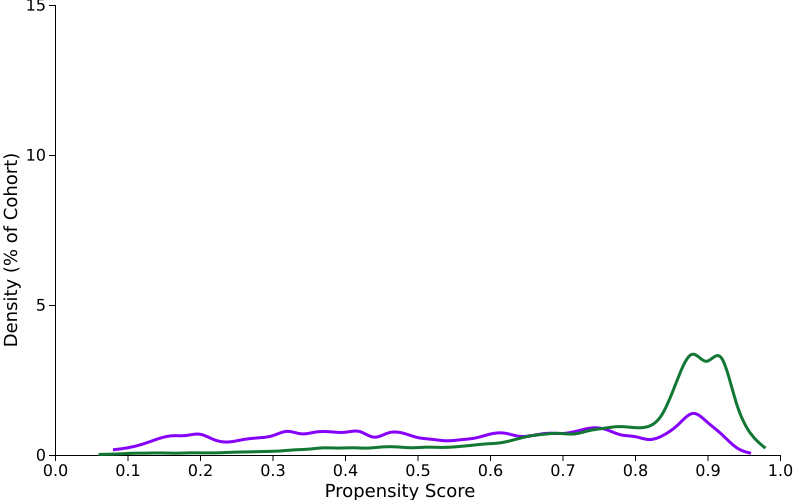 | 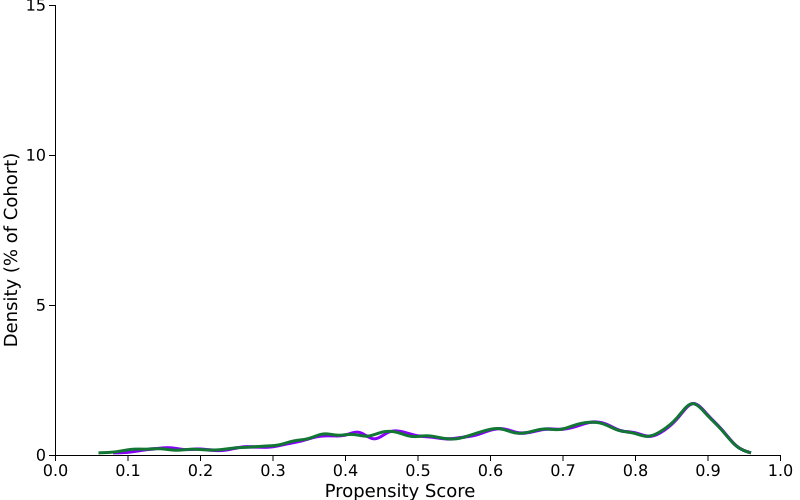 |

Kernel density plots of propensity score distributions for patients with sickle cell disease and type 2 diabetes mellitus treated with GLP-1 receptor agonists (purple) and those not treated with GLP-1 therapy (green), shown before and after propensity score matching. After matching, substantial overlap in propensity score distributions demonstrate improved covariate balance between cohorts.
